# Supplementary material for: Resolving the full spectrum of human genome variation using Linked-Reads
Source: Genome Res. 2019 Apr;29(4):635–45. doi: 10.1101/gr.234443.118 (PMC6442396; doi:10.1101/gr.234443.118)
Supplement: Supplemental Material [file supp_gr.234443.118_Supplemental_longranger-2.2.zip › longranger-2.2.tar.bz2!/lr2.2-public/lib/stan/cmdstan/stan/doc/api/html/agrad_2fwd_2matrix_2sort_8hpp_source.html]

Stan: src/stan/agrad/fwd/matrix/sort.hpp Source File


|  |  |
| --- | --- |
|  | Stan  2.5.0  probability, sampling & optimization |


- Main Page
- Related Pages
- Namespaces
- Classes
- Files

- File List
- File Members

All Classes Namespaces Files Functions Variables Typedefs Enumerations Enumerator Friends Macros Pages

- src
- stan
- agrad
- fwd
- matrix

sort.hpp

Go to the documentation of this file.

1 #ifndef STAN\_\_AGRAD\_\_FWD\_\_SORT\_HPP

2 #define STAN\_\_AGRAD\_\_FWD\_\_SORT\_HPP

3

4 #include <stan/agrad/fwd/fvar.hpp>

5 #include <stan/meta/traits.hpp>

6 #include <vector>

7 #include <stan/math/matrix/Eigen.hpp>

8 #include <algorithm> // std::sort

9 #include <functional> // std::greater

10

11 namespace stan {

12

13  namespace agrad {

14

15  template <typename T>

16  inline

17  std::vector< fvar<T> >

18  sort\_asc(std::vector< fvar<T> > xs) {

19  std::sort(xs.begin(), xs.end());

20  return xs;

21  }

22

23  template <typename T>

24  inline

25  std::vector< fvar<T> >

26  sort\_desc(std::vector< fvar<T> > xs) {

27  std::sort(xs.begin(), xs.end(), std::greater< fvar<T> >());

28  return xs;

29  }

30

31  template <typename T, int R, int C>

32  inline

33  typename Eigen::Matrix<fvar<T>,R,C>

34  sort\_asc(Eigen::Matrix<fvar<T>,R,C> xs) {

35  std::sort(xs.data(), xs.data()+xs.size());

36  return xs;

37  }

38

39  template <typename T, int R, int C>

40  inline

41  typename Eigen::Matrix<fvar<T>,R,C>

42  sort\_desc(Eigen::Matrix<fvar<T>,R,C> xs) {

43  std::sort(xs.data(), xs.data()+xs.size(), std::greater< fvar<T> >());

44  return xs;

45  }

46

47  }

48 }

49 #endif

stan::agrad::fvar

**Definition:** fvar.hpp:13

traits.hpp

stan::agrad::sort\_asc

std::vector< fvar< T > > sort\_asc(std::vector< fvar< T > > xs)

**Definition:** sort.hpp:18

fvar.hpp

stan::agrad::sort\_desc

std::vector< fvar< T > > sort\_desc(std::vector< fvar< T > > xs)

**Definition:** sort.hpp:26

Eigen.hpp


---

[ Stan Home Page ]

*© 2011–2014,
Stan Development Team.*
